# CD4-Independent Human Immunodeficiency Virus Infection Involves Participation of Endocytosis and Cathepsin B

**DOI:** 10.1371/journal.pone.0019352

**Published:** 2011-04-25

**Authors:** Hiroaki Yoshii, Haruka Kamiyama, Kensuke Goto, Kazunori Oishi, Nobuhiko Katunuma, Yuetsu Tanaka, Hideki Hayashi, Toshifumi Matsuyama, Hironori Sato, Naoki Yamamoto, Yoshinao Kubo

**Affiliations:** 1 Department of AIDS Research, Institute of Tropical Medicine, Global Center of Excellence (GCOE), Nagasaki University, Nagasaki, Japan; 2 Department of Eco-epidemiology, Institute of Tropical Medicine, Nagasaki University, Nagasaki, Japan; 3 Department of Preventive and Therapeutic Research for Infectious Diseases, Graduate School of Biomedical Sciences, Nagasaki University, Nagasaki, Japan; 4 International Research Center for Infectious Diseases, Research Institute for Microbial Diseases, Osaka University, Osaka, Japan; 5 Institute for Health Sciences, Tokushima Bunri University, Tokushima, Japan; 6 Department of Immunology, Graduate School and Faculty of Medicine, University of the Ryukyus, Nishihara, Okinawa, Japan; 7 Division of Cytokine Signaling, Graduate School of Biomedical Sciences, Nagasaki University, Nagasaki, Japan; 8 Laboratory of Viral Genomics, Pathogen Genomics Center, National Institute of Infectious Diseases, Tokyo, Japan; 9 AIDS Research Center, National Institute of Infectious Diseases, Tokyo, Japan; University of Nebraska Medical Center, United States of America

## Abstract

During a comparison of the infectivity of mNDK, a CD4-independent human immunodeficiency virus type 1 (HIV-1) strain, to various cell lines, we found that HeLa cells were much less susceptible than 293T and TE671 cells. Hybridoma cells between HeLa and 293T cells were as susceptible as 293T cells, suggesting that cellular factors enhance the mNDK infection in 293T cells. By screening a cDNA expression library in HeLa cells, cystatin C was isolated as an enhancer of the mNDK infection. Because cathepsin B protease, a natural ligand of cystatin C, was upregulated in HeLa cells, we speculated that the high levels of cathepsin B activities were inhibitory to the CD4-independent infection and that cystatin C enhanced the infection by impairing the excessive cathepsin B activity. Consistent with this idea, pretreatment of HeLa cells with 125 µM of CA-074Me, a cathepsin B inhibitor, resulted in an 8-fold enhancement of the mNDK infectivity. Because cathepsin B is activated by low pH in acidic endosomes, we further examined the potential roles of endosomes in the CD4-independent infection. Suppression of endosome acidification or endocytosis by inhibitors or by an Eps15 dominant negative mutant reduced the infectivity of mNDK in which CD4-dependent infections were not significantly impaired. Taken together, these results suggest that endocytosis, endosomal acidification, and cathepsin B activity are involved in the CD4-independent entry of HIV-1.

## Introduction

Human immunodeficiency virus type 1 (HIV-1) gains entry into host cells by fusion of the viral envelope membrane with the host cell membrane. This process is generally initiated by binding of the HIV-1 envelope glycoprotein gp120 to CD4 on the host cell surface. The binding then induces conformational changes of the gp120, which allows the gp120 to interact with a cellular surface chemokine receptor, termed coreceptor [1]. HIV-1 can use many types of chemokine receptors as the coreceptors [2], but the two most common types of coreceptors for HIV-1 entry are CXCR4 and CCR5.

HIV-1 variants that do not require CD4 for infection have been isolated in vitro [3], [4], [5] and in vivo [6], [7]. Gp120 coreceptor binding sites of CD4-independent HIV-1 variants are exposed before the CD4 binding, and the CD4-independent gp120 directly interacts with the coreceptor for the entry [5]. It has been reported that CD4-negative cells such as liver, kidney, and CD8+ T cells are infected with the CD4-independent HIV-1 in AIDS patients, and such CD4-independent variants are thought to be associated with hepatitis, nephropathy, and CD8+ T cell dysfunction in AIDS patients [6], [8], [9], [10]. Almost all simple retroviruses, including murine leukemia viruses (MLVs), recognize multiple membrane-spanning proteins as the HIV-1 coreceptors. CD4-independent variants of simian immunodeficiency virus have been isolated more frequently than CD4-independent HIV-1 [11], [12]. HIV-1 variants that recognize CD4 as a sole receptor have not been isolated. These results suggest that CD4-independent HIV-1 variants are prototypes of CD4-dependent strains.

Inhibitors of endosome acidification attenuate infections by many retroviruses, including MLV, avian leukosis virus, Jaagsiekte sheep retrovirus, equine infectious anemia virus, and foamy virus [13], [14], [15], [16], [17], [18], [19], [20]. It has recently been reported that inhibitors of endosomal cathepsin proteases attenuate ecotropic MLV infection [19], [20]. These results indicate that the entry of these retroviruses occurs through acidic late endosomes and requires endosomal cathepsin proteases, such as Ebola virus, reovirus, Japanese encephalitis virus, and coronavirus [21], [22], [23], [24]. Because cathepsin proteases are activated by low pH in acidic endosomes, the endosome acidification inhibitors might attenuate the virus infections by suppressing cathepsin protease activation. However, the endosome acidification inhibitors do not suppress CD4-dependent HIV-1 infections, but rather enhance them [25]. Therefore, the CD4-dependent HIV-1 entry likely occurs at the host cell surface, but not through endosomes. However, it has recently been shown that CD4-dependent HIV-1 enters into host cells via endosomes [26], [27]. Due to these conflicting observations, it is unclear whether the CD4-dependent HIV-1 entry occurs through endosomes or through direct fusion at the cell surface membrane.

The CD4-independent mNDK HIV-1 strain was isolated by adaptation of the parental CD4-dependent CXCR4-tropic NDK virus to CD4-negative cells [4]. The CD4-independent mNDK variant can infect and induce syncytia in CD4-negative CXCR4-positive cells. However, the mNDK virus more efficiently infects CD4-positive cells than CD4-negative cells, suggesting that the mNDK virus induces CD4-independent and -dependent infections in CD4-negative and -positive cells, respectively [28].

In the present study, we found that HeLa cells are much less susceptible to infection by an HIV-1 vector having the mNDK virus envelope protein (Env) than 293T cells. Hybridoma cells between HeLa and 293T cells were as susceptible to the mNDK vector infection as 293T cells, indicating that HeLa cells lack a cellular factor(s) required for the CD4-independent mNDK vector infection. We aimed to identify the cellular factor by screening of a cDNA expression library prepared from human lymph nodes. Cystatin C, which inhibits cystein cathepsin proteases, was isolated as an enhancer of the CD4-independent mNDK vector infection in HeLa cells. A synthetic cathepsin B inhibitor, CA-074Me, also enhanced the CD4-independent mNDK vector infection in HeLa cells, but did not affect the CD4-dependent infection. These results show that cathepsin B inhibits the CD4-independent HIV-1 vector infection. Because cathepsin proteases are activated by low pH in acidic late endosomes, the CD4-independent mNDK infection might occur through endosomes. Inhibitors of endosome acidification or endocytosis significantly attenuated the CD4-independent HIV-1 vector infection, but not the CD4-dependent infection, as expected. These results suggest that the CD4-independent HIV-1 enters through acidic late endosomes but the CD4-dependent HIV-1 does not.

## Results

### HeLa cells are much less susceptible to CD4-independent mNDK HIV-1 vector infection than 293T cells

**Figure 1:**
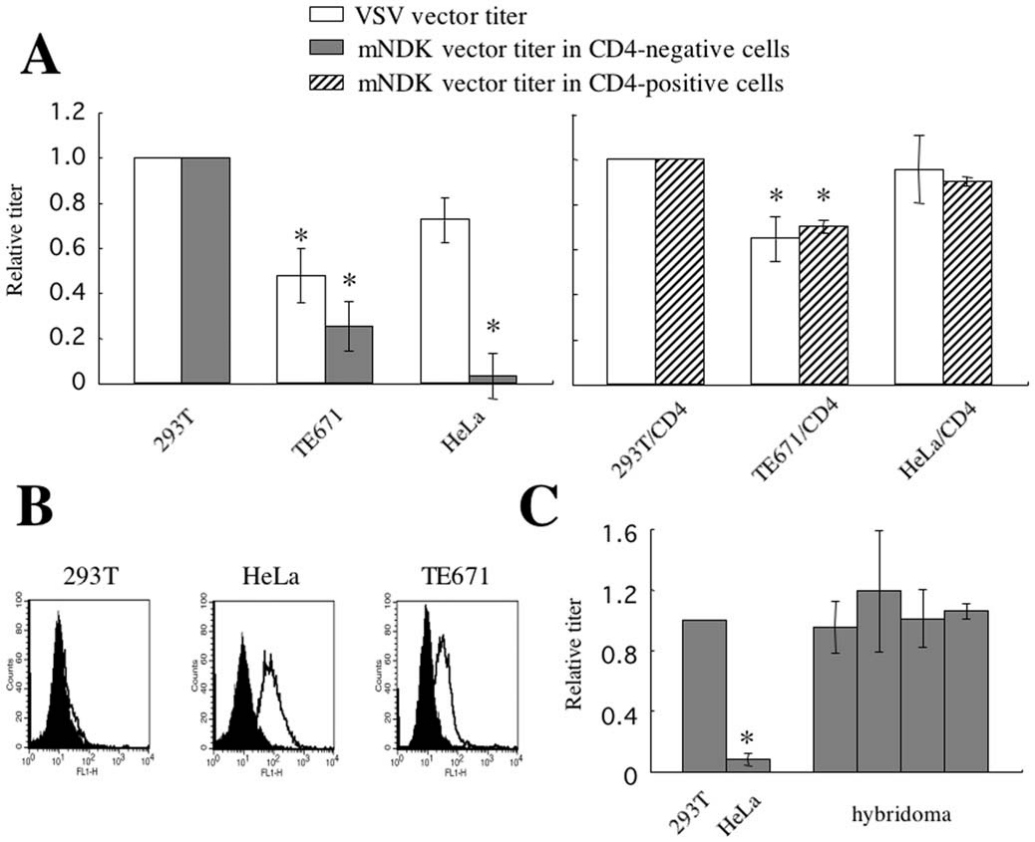
Susceptibility of 293T, TE671, and HeLa cells to CD4-independent mNDK HIV-1 vector infection. (A) Transduction titers of VSV-G- or mNDK Env protein-pseudotyped HIV-1 vector on 293T, TE671, HeLa, and their CD4-expressing cells were measured. Relative values to the transduction titer on 293T (left panel) or 293T/CD4 cells (1×10^5^) (right panel) are indicated. This experiment was repeated 3 times, and the results are shown as the means +− SD. Asterisks indicate statistically significant differences compared to the relative value on the control cells. (B) The cell surface expression of CXCR4 was analyzed on 293T, TE671, and HeLa cells by a fluorescence flow cytometer using an anti-CXCR4 monoclonal antibody. Closed and open areas indicate cells treated only with the secondary FITC-conjugated anti-rat IgG antibody and cells treated with the CXCR4 antibody and then with the secondary antibody, respectively. (C) Transduction titers of the mNDK HIV-1 vector on hybridoma cells between 293T and HeLa cells were measured. Relative values to the transduction titer on 293T cells are indicated. This experiment was repeated 3 times, and the results are shown as the means +− SD. Asterisks indicate statistically significant differences compared to the relative value on 293T cells.

To assess the susceptibility of 293T, TE671, and HeLa cells to an HIV-1 vector containing the CD4-independent mNDK Env protein, transduction titers of the HIV-1 vector were measured in these cells. Transduction titers of the VSV-G- and mNDK Env-bearing HIV-1 vectors in TE671 cells were about 50% and 30% of those in 293T cells, respectively (Fig. 1A, left panel). When normalized by the titer of VSV vector, susceptibility of TE671 cells to the CD4-independent mNDK vector infection was about 60% of that in 293T cells. Transduction titers of the VSV and mNDK vectors in HeLa cells were about 70% and 3% of those in 293T cells, respectively. Normalization by the titer of VSV vector indicated that the susceptibility of HeLa cells to the CD4-independent mNDK vector infection was only about 4% of that in 293T cells. However, when these cells were engineered to express CD4, HeLa/CD4 cells were found to be as susceptible to the CD4-dependent mNDK vector infection as 293T/CD4 and TE671/CD4 cells (Fig. 1A, right panel). Because CXCR4 is the infection receptor for the CD4-indepndent infection of the mNDK vector [4], [28], cell surface expressions of CXCR4 in these cells were analyzed. As the result, HeLa cells expressed CXCR4 more efficiently than 293T cells (Fig. 1B). These results indicate that HeLa cells either possess an inhibitory factor(s) or lack a factor(s) necessary for the CD4-independent mNDK HIV-1 vector infection.

### HeLa cells lack a cellular factor(s) required for CD4-independent HIV-1 vector infection

To determine whether “positive” or “negative” factors are involved in the resistant phenotype of HeLa cells, hybridoma cells between HeLa and 293T cells were constructed. HeLa cells were inoculated with a puromycin resistance gene-encoding MLV vector to construct drug-resistant cells. 293T cells are originally geneticin-resistant. The puromycin-resistant HeLa cells were fused with the geneticin-resistant 293T cells by polyethylene glycol, and hybridoma cells were obtained by co-selection with puromycin and geneticin. The double antibiotics-resistant cell pools were used for the following study. All four independent hybridoma cell pools were as susceptible to the CD4-independent mNDK vector infection as 293T cells (Fig. 1C). This shows that HeLa cells lack a cellular factor(s) that is required for the CD4-independent mNDK vector infection.

### Identification of cystatin C as a crucial factor for the CD4-independent mNDK vector infection

**Figure 2:**
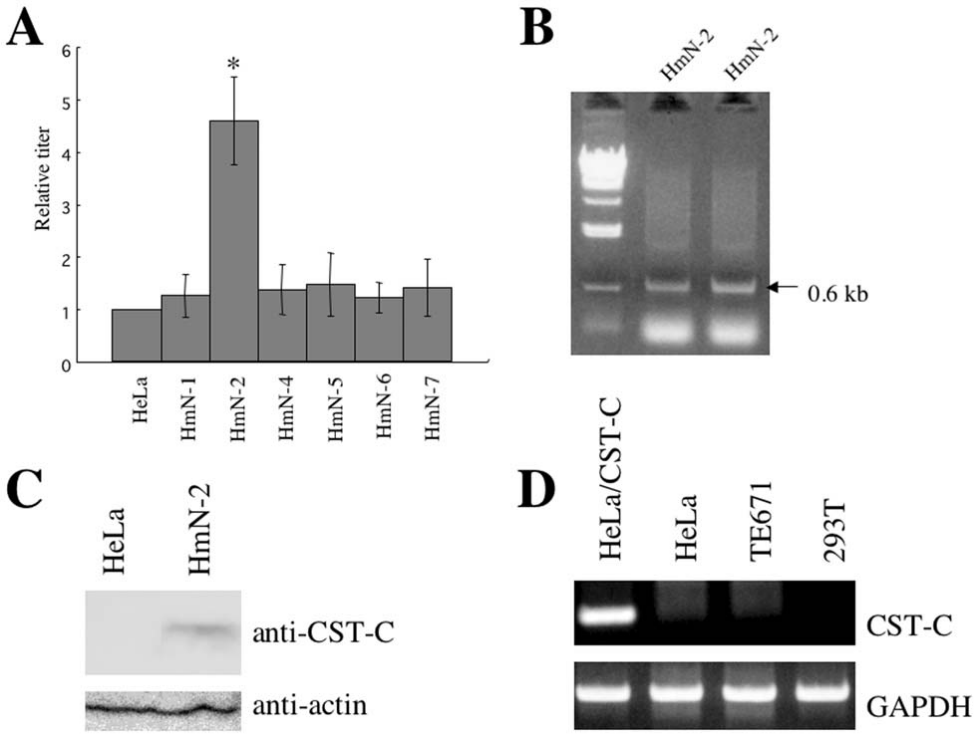
Cystatin C was isolated as an enhancer of the CD4-independent mNDK HIV-1 vector infection. (A) A blasticidin resistance gene-bearing mNDK HIV-1 vector was inoculated into the cDNA expression library-transfected HeLa cells, and blasticidin-resistant cell clones were isolated. Transduction titers of the LacZ gene-bearing mNDK HIV-1 vector were measured on these blasticidin-resistant cell clones. Relative values to the transduction titer on normal HeLa cells are indicated. This experiment was repeated 3 times, and the results are shown as the means +− SD. Asterisks indicate statistically significant differences compared to the relative value on HeLa cells. (B) PCR was performed using genomic DNA samples prepared from the HmN-2 cells. The PCR products were subjected to agarose gel electrophoresis. (C) A cell lysate prepared from the HmN-2 cells was analyzed by Western immunoblotting using an anti-cystatin C (upper panel) or anti-actin (lower panel) antibody. (D) To detect cystatin C or GAPDH mRNA, RT-PCR was performed. The PCR products were subjected to agarose gel electrophoresis.

To identify the cellular factor required for the CD4-independent mNDK vector infection, HeLa cells were transfected with a cDNA expression library constructed from human lymph nodes, because it has been reported that human lymph node cells including CD8+ T-lymphocytes are highly susceptible to CD4-independent HIV-1 variants [29]. Because the plasmid vector used in the cDNA library construction contains a neomycin-resistance gene, the transfected HeLa cells were selected by geneticin. The geneticin-resistant HeLa cells were inoculated with an mNDK HIV-1 vector containing a blasticidin-resistance gene as a selectable marker. The mNDK HIV-1 vector was diluted to obtain one blasticidin-resistant cell colony in normal HeLa cells per a 10-cm culture dish. This experiment was repeated seven times, and finally six blasticidin-resistant cell clones were obtained. Among these blasticidin-resistant cell clones, one cell clone (HmN-2) was 4.5 times more susceptible to the LacZ gene-encoding mNDK HIV-1 vector infection than parental HeLa cells (Fig. 2A). The susceptibility of the other blasticidin-resistant cell clones was almost same as that of the original HeLa cells.

To obtain the cDNA fragment expressed in the HmN-2 cells, PCR was performed using a genomic DNA prepared from the HmN-2 cells. The PCR primers were complementary to both flanking regions of the cDNA inserts in the cDNA library plasmid. As a result, an approximately 0.6-kb PCR fragment was detected (Fig. 2B), and partial sequencing indicated that it was cystatin C. The endogenous cystatin C mRNA is about 0.7-kb long, and encodes a 120-amino acid protein. The cystatin C protein is abundantly expressed in many tissues, and inhibits cystein protease activity [30]. Expression of the cystatin C protein in the HmN-2 cells was analyzed by Western immunoblotting using an anti-cystatin C antibody (Fig. 2C). The cystatin C mRNA was not detected in HeLa, TE671, and 293T cells (Fig. 2D).

**Figure 3:**
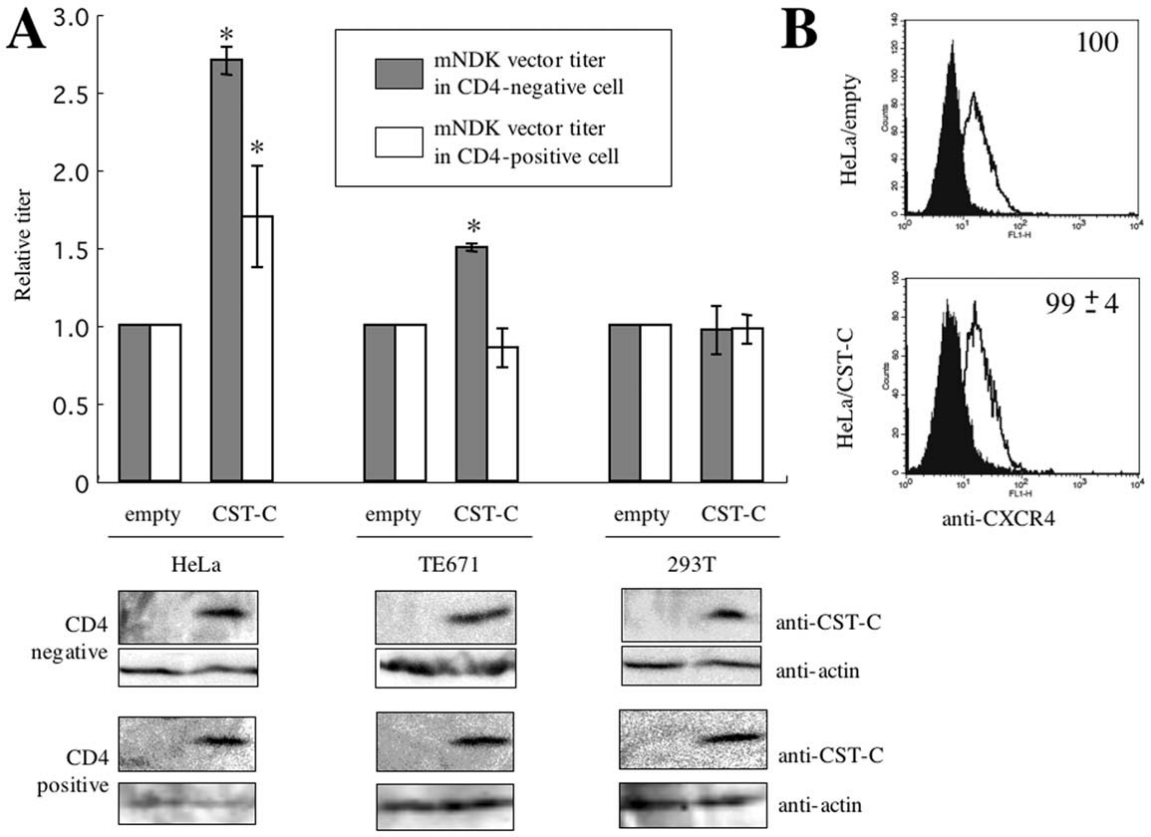
Cystatin C enhances the CD4-independent mNDK HIV-1 vector infection. (A) Transduction titers of the mNDK HIV-1 vector were measured on cystatin C-transduced and -untransduced HeLa, TE671, 293T, and their CD4-expressing cells. Relative values to the transduction titer on cystatin C-untransduced cells are indicated (upper panel). This experiment was repeated 3 times, and the results are shown as the means +− SD. Asterisks indicate statistically significant differences compared to the relative value on the each cystatin C-untransduced cells. Cystatin C expression was confirmed by Western immunoblotting using the cystatin C antibody (lower panel). (B) The cell surface expression of CXCR4 on the cystatin C-transduced and –untransduced HeLa cells was analyzed by a fluorescence flow cytometer. Closed and open areas indicate cells treated with control serum and then with the secondary FITC-conjugated anti-rat IgG antibody and cells treated with the CXCR4 antibody and then with the secondary antibody, respectively. Relative values to means of fluorescence intensity in the untransduced cells are also indicated. This experiment was repeated 3 times, and the results are shown as averages +− SD.

To confirm the role of cystatin C in the CD4-independent mNDK vector infection, HeLa, HeLa/CD4, TE671, TE671/CD4, 293T, and 293T/CD4 cells stably expressing cystatin C were constructed by using an MLV vector encoding cystatin C. HeLa cells expressing cystatin C were about 3 times more susceptible to the mNDK vector infection than control HeLa cells transduced by the empty MLV vector (Fig. 3A, upper panels). Cystatin C expression also enhanced the CD4-dependent infection, but the effect on the CD4-dependent infection was less than that on the CD4-independent infection. Cystatin C protein expression in these transduced cells was confirmed by Western immunoblotting (Fig. 3A, lower panels). Cystatin C expression resulted in a 1.5-fold enhancement of the susceptibility of TE671 cells to the CD4-independent HIV-1 vector infection, but did not affect the susceptibility of TE671/CD4 cells. Cystatin C expression did not affect the mNDK vector infection in CD4-negative and –positive 293T cells. Cystatin C did not affect the cell surface expression of CXCR4 in HeLa cells (Fig. 3B), indicating that cystatin C does not increase the CD4-independent vector infection by enhancing CXCR4 expression. Collectively, these findings indicate that cystatin C enhances the CD4-independent mNDK vector infection in HeLa and TE671 cells, but does not in 293T cells.

### Cellular susceptibility to CD4-independent infection is reverse-correlated with cathepsin B activity

**Figure 4:**
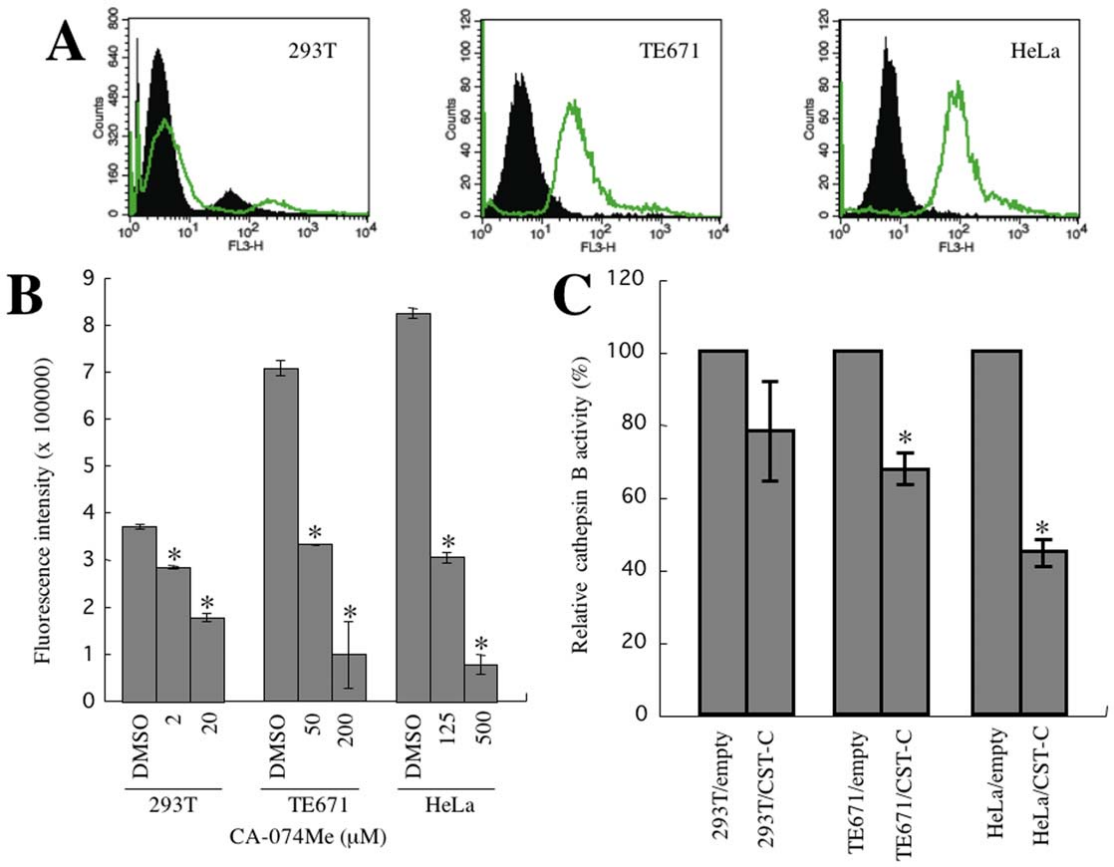
Cathepsin B activity of 293T, TE671, and HeLa cells. (A) Cathepsin B activities in living 293T, TE671, and HeLa cells were analyzed by the cathepsin B detection reagent. The closed area and green line indicate cells unstained and cells stained with the cathepsin B detection reagent, respectively. (B) Fluorescence intensities of cell lysates prepared from HeLa, TE671, 293T, and their CA-074Me-treated cells were measured to quantitate cathepsin B activity by using a cathepsin B activity assay kit. This experiment was repeated 3 times, and the results are shown as the means +− SD. Asterisks indicate statistically significant differences compared to the fluorescence intensity on DMSO-treated control cells. (C) Cathepsin B activities of cell lysates prepared from HeLa, TE671, 293T, and their cystatin C-expressing cells were analyzed by using a cathepsin B activity assay kit. Relative values to the fluorescence intensity for each cell type not expressing cystatin C are indicated. This experiment was repeated 3 times, and the results are shown as the means +− SD. Asterisks indicate statistically significant differences compared to the relative value on the cells not expressing cystatin C.

Next, we quantitatively measured cathepsin B activity in HeLa, TE671, and 293T cells (see Materials and Methods). The cathepsin B activities were highest in HeLa cells, followed in order by TE671 and 293T cells (Figs. 4A and B). The target cells expressing higher level of cathepsin B activity were less susceptible to the CD4-independent mNDK vector infection, suggesting that cathepsin B inhibits the CD4-independent HIV-1 infection. Cystatin C expression indeed reduced cathepsin B activities in TE671 and HeLa cells (Fig. 4C). Cystatin C neither inhibited cathepsin B activity nor enhanced the CD4-independent vector infection in 293T cells (Fig. 3A), probably because the cathepsin B activity of 293T cells was originally low.

### A synthetic cathepsin B inhibitor enhances susceptibility to CD4-independent infection

**Figure 5:**
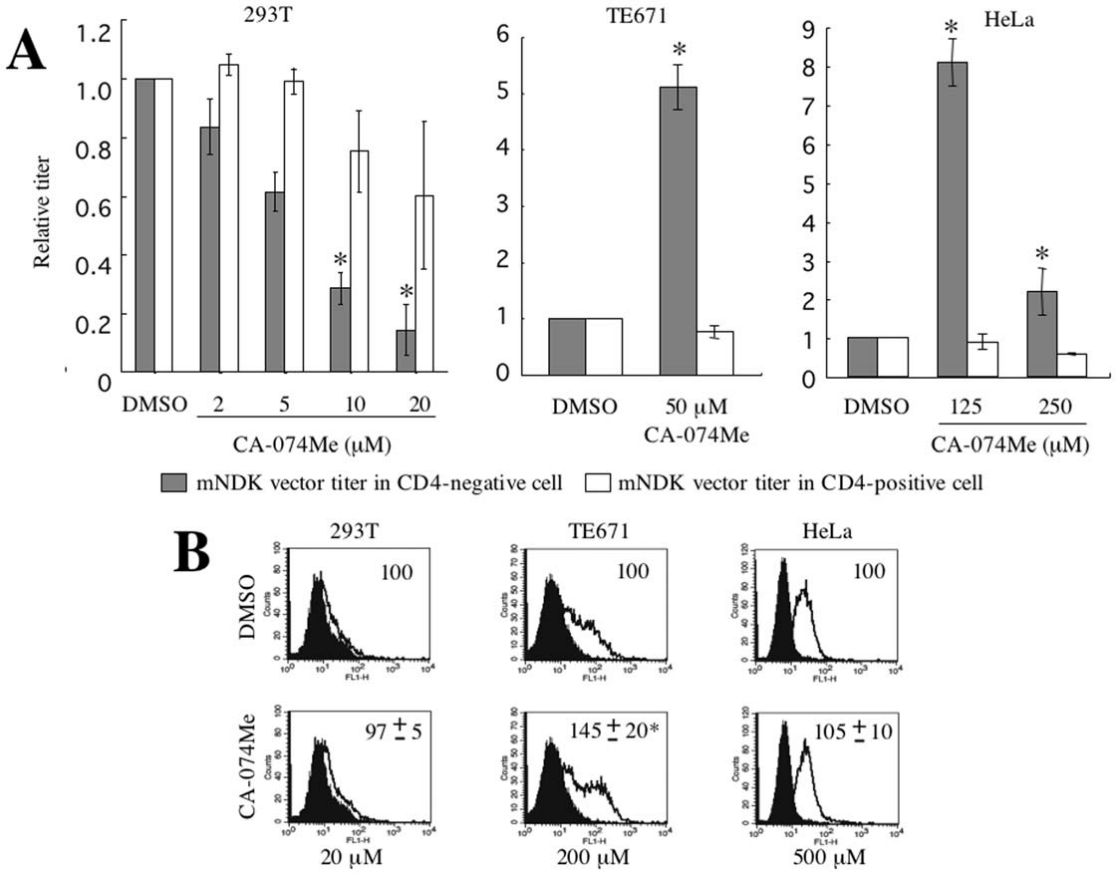
Effects of a cathepsin B inhibitor, CA-074Me, on the CD4-independent HIV-1 vector infection. (A) Transduction titers were measured on CA-074Me- or DMSO-treated 293T, TE671, and HeLa cells. Relative values to the titer on cells treated with an equal volume of DMSO are indicated. This experiment was repeated 3 times, and the results are shown as the means +− SD. Asterisks indicate statistically significant differences compared to the relative value on each type of DMSO-treated cells. (B) The cell surface expression of CXCR4 was analyzed on CA-074Me-treated cells. Closed and open areas indicate cells treated with the control serum and then with the secondary FITC-conjugated anti-rat IgG antibody and cells treated with the CXCR4 antibody and then with the secondary antibody, respectively. Relative values to means of fluorescence intensity in the DMSO-treated cells are also indicated. This experiment was repeated 3 times, and the results are shown as averages +− SD.

To address whether cystatin C enhances the CD4-independent HIV-1 vector infection by inhibiting cathepsin protease activity, the effects of synthetic cathepsin protease inhibitors on CD4-independent vector infection were analyzed. Target cells were pretreated with cathepsin inhibitors for 5 h. As expected, when HeLa cells were treated with a specific cathepsin B inhibitor, CA-074Me, at 125 µM, the transduction titer of the mNDK vector was increased 8-fold (Fig. 5A). Similarly, CA-074Me treatment of TE671 cells at 50 µM induced a 5-fold enhancement of the CD4-independent infection. In association with this change, cathepsin B activity was indeed reduced by these CA-074Me treatments in HeLa and TE671 cells to about 50%, a level similar to that in the control 293T cells (Fig. 4B). These results indicate that cathepsin B inhibition by cystatin C or CA-074Me leads to enhancement of the CD4-independent vector infection, suggesting that cathepsin B plays a role in suppressing the CD4-independent HIV-1 infection in HeLa and TE671 cells, which express cathepsin B at a relatively higher level.

In contrast, CA-074Me treatment attenuated the CD4-independent vector infection in a dose-dependent manner in 293T cells (Fig. 5A), whose cathepsin B activity was extremely low (Figs. 4A and B). The 293T cells were then treated with CA-074Me at a lower concentration to see whether or not the CD4-independent vector infection would be promoted. By the CA-074Me treatment of 293T cells at 2 µM, cathepsin B activity was only slightly inhibited, to about 85% of that in DMSO-treated 293T cells. But when 293T cells were treated with CA-074Me at a lower concentration, cathepsin B activity in the treated 293T cells was not affected and the CD4-independent infection was not enhanced. The CA-074Me treatment did not affect the CD4-dependent mNDK vector infection in 293T/CD4 cells.

The cathepsin L specific inhibitor, CLIK148 [31], did not affect the mNDK vector infection in CD4-negative and –positive cells (data not shown).

### SAOS-2 cells are resistant to the CD4-independent infection due to high cathepsin B activity

**Figure 6:**
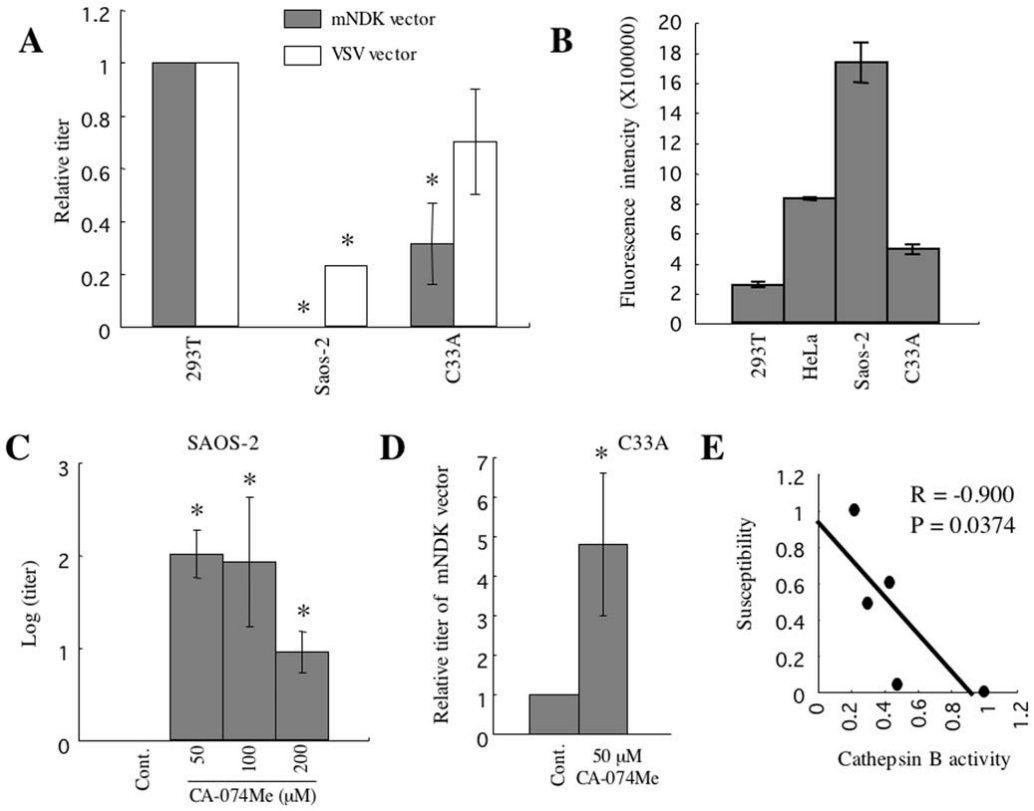
SAOS-2 cells are resistant to the CD4-independent HIV-1 vector infection. (A) Transduction titers of the mNDK and VSV-G vectors were measured on 293T, SAOS-2, and C33A cells. Relative values to the titers on 293T cells are indicated. (B) Cathepsin B activities of cell lysates from 293T, HeLa, SAOS-2 and C33A cells were measured by the cathepsin B activity assay kit. (C) Transduction titers of the mNDK vector were measured on DMSO- (Cont.) or CA-074Me-treated SAOS-2 cells. (D) Transduction titers of the mNDK vector were measured on DMSO- (Cont.) or CA-074Me-treated C33A cells. These experiments were repeated 3 times, and the results are shown as the means +− SD. Asterisks indicate statistically significant differences compared to the control cells. (E) Relative values to the cathepsin B activity of SAOS-2 cells (x axis) and to the transduction titer in 293T cells (y axis) are plotted. Correlation between cathepsin B activity and susceptibility to the CD4-independent infection was analyzed by Spearman's rank correlation.

The above results suggest that cathepsin B activity is reverse-correlated with susceptibility to the CD4-independent mNDK vector infection in 293T, TE671, and HeLa cells. To further confirm the result, susceptibility to the CD4-independent infection and cathepsin B activity of other two cell lines, SAOS-2 and C33A cells, were measured. Surprisingly, mNDK vector-infected cells were not detected in SAOS-2 cells (Fig. 6A). Cathepsin B activity of SAOS-2 cells was higher than that of HeLa cells (Fig. 6B), and CA-074Me treatment conferred SAOS-2 cells susceptible to the CD4-independent mNDK vector infection (Fig. 6C), suggesting that SAOS-2 cells are completely resistant to the CD4-independent infection due to the high level of cathepsin B activity. However, because SAOS-2 cells were less susceptible to the VSV-G-pseudotyped HIV-1 vector infection than 293T cells (Fig. 6A), the infection resistance of SAOS-2 cells is likely to involve post-entry mechanisms either. C33A cells were as susceptible to the CD4-independent mNDK vector infection as TE671 cells, and have similar level of cathepsin B activity to TE671 cells (Figs. 6A and B). CA-074Me treatment of C33A cells at 50 µM enhanced the CD4-independent infection about 5 times, like TE671 cells (Fig. 6D). Statistical analysis reveals that susceptibility to the CD4-independent mNDK vector infection was reverse-correlated with cathepsin B activity of the target cells (Fig. 6E). These results strongly support the conclusion that cathepsin B is one of the critical cellular factors determining susceptibility to the CD4-independent HIV-1 infection.

### Expression of cathepsin B results in inhibition of CD4-independent infection in 293T cells

**Figure 7:**
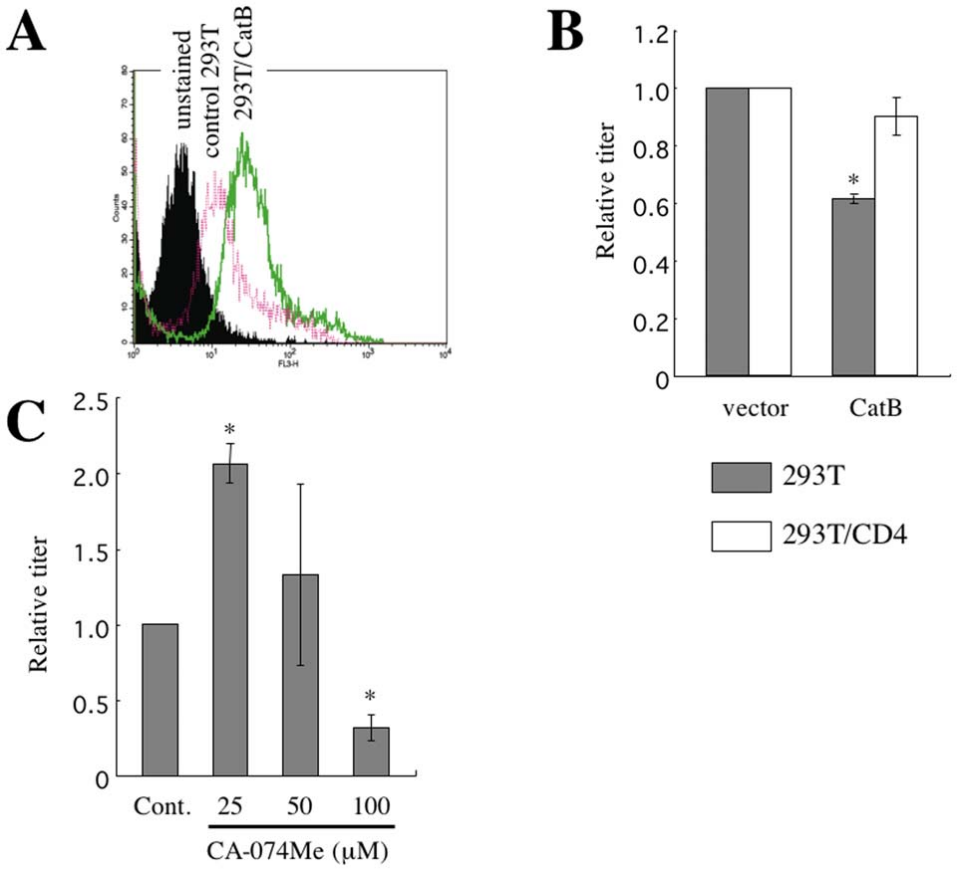
Cathepsin B attenuates the CD4-independent HIV-1 vector infection. (A) Cathepsin B activities of control and cathepsin B-overexpressing 293T cells were measured by the cathepsin B detection reagent. The closed area indicates cells not stained with the cathepsin B detection reagent. The red and green lines indicate the control and cathepsin B-overexpressing 293T cells, respectively. (B) Transduction titers of the mNDK vector were measured on cathepsin B-untransduced and –transduced 293T and 293T/CD4 cells. Relative values to the titers on the untransduced 293T and 293T/CD4 cells are indicated. (C) Transduction titers of the mNDK vector were measured on CA-074Me-treated, cathepsin B-overexpressing 293T cells. Relative values to the titer on DMSO-treated cells are indicated. These experiments were repeated 3 times, and the results are shown as the means + SD. Asterisks indicate statistically significant differences compared to the relative value on the control cells.

The above results indicate that cathepsin B inhibits the CD4-independent mNDK vector infection in cells with high cathepsin B activity. To confirm that cathepsin B inhibits the CD4-independent vector infection, the effects of cathepsin B overexpression on the CD4-independent vector infection were analyzed in 293T cells, in which cathepsin B is expressed at a low level. 293T cells were inoculated with an MLV vector encoding cathepsin B and puromycin-resistance genes, and were selected by puromycin. The puromycin-resistant 293T cell pool indeed had elevated level of cathepsin B activity compared to the control 293T cells (Fig. 7A). The cathepsin B overexpression significantly inhibited the CD4-independent vector infection, but did not the CD4-dependent infection (Fig. 7B). When the cathepsin B-overexpressing 293T cells were treated with CA-074Me at 25 µM, the CD4-independent vector infection was enhanced (Fig. 7C), as in HeLa and TE671 cells. These results suggest that cathepsin B is a host defense factor against the CD4-independent infection, but not against the CD4-dependent infection.

### Cathepsin B functions as an innate immune factor against CD4-independent HIV-1 infection

It has been reported that LPS treatment of cells enhances cathepsin B expression [32], [33], [34]. Therefore, the activation of innate immunity by LPS-mediated TLR4 signaling might attenuate the CD4-independent HIV-1 infection. To address this hypothesis, 293T cells originally expressing cathepsin B at a low level (Fig. 4A) were transfected by a CD4-TLR4 expression plasmid constructed by our research group. CD4-TLR4 is a fusion protein of the extracellular domain of CD4 and the intracellular domain of TLR4, and functions as a constitutively active mutant of TLR4 [35]. NF- B activation by the transfection of 293T cells with the CD4-TLR4 expression plasmid was observed (data not shown). Because the extracellular domain of CD4-TLR4 was derived from mouse CD4, it should not directly affect the CD4-independent HIV-1 vector infection [36].

**Figure 8:**
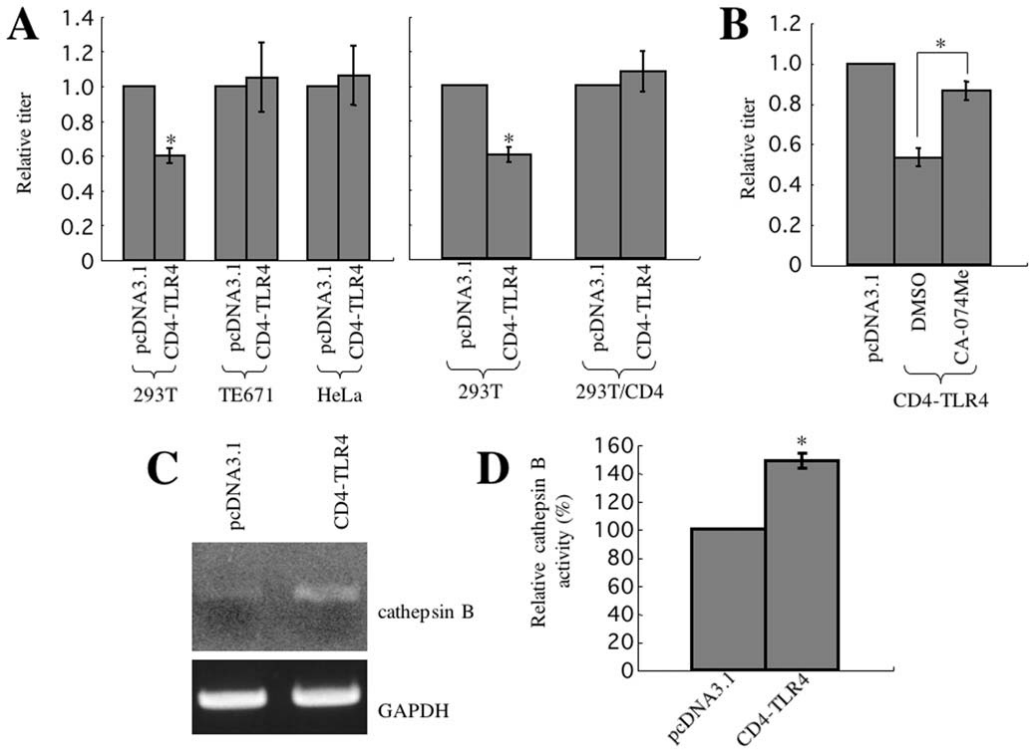
TLR4 signal activation attenuates the CD4-independent HIV-1 vector infection by enhancing cathepsin B expression. (A) Transduction titers of the mNDK HIV-1 vector were measured on pcDNA3.1- or CD4-TLR4 expression plasmid-transfected 293T, TE671, HeLa, (left panel) and 293T/CD4 (right panel) cells. Relative values to the titer on the pcDNA3.1-transfected cells are indicated. (B) Transduction titers of the mNDK vector were measured on CA-074Me- or DMSO-treated CD4-TLR4-transfected 293T cells. Relative values to the titer on the pcDNA3.1-transfected 293T cells are shown. These experiments were repeated 3 times, and the results are shown as the means +− SD. Asterisks indicate statistically significant differences compared to the relative value on the control cells. (C) Cathepsin B mRNA expression was analyzed by semi-quantitative RT-PCR. PCR products were subjected to agarose gel electrophoresis. (D) The cathepsin B activity of the pcDNA3.1- or CD4-TLR4 expression plasmid-transfected 293T cells was measured by a cathepsin B activity assay kit. Relative values to the fluorescence intensity on the pcDNA3.1-transfected cells are indicated. This experiment was repeated 3 times, and the results are shown as the means +− SD. Asterisks indicate statistically significant differences compared to the relative value on the pcDNA3.1-transfected cells.

As expected, TLR4 signal activation by CD4-TLR4 reduced the CD4-independent vector infection in 293T cells (Fig. 8A, left panel). The CD4-TLR4 transfection did not affect the CD4-independent infection in TE671 and HeLa cells, in which cathepsin B was originally expressed at a high level. The CD4-TLR4 transfection did not affect the CD4-dependent HIV-1 vector infection in 293T/CD4 cells (Fig. 8A, right panel). The inhibitory effect of CD4-TLR4 on the CD4-independent infection was abrogated by treatment with the cathepsin B inhibitor, CA-074Me (10 µM) (Fig. 8B). CD4-TLR4 plasmid transfection indeed enhanced cathepsin B mRNA expression as analyzed by RT-PCR (Fig. 8C), and cathepsin B activity 1.5-fold in 293T cells (Fig. 8D). These results indicate that the TLR4 signal activation induces cathepsin B expression and renders the cells resistant to CD4-independent HIV-1 infection, suggesting that cathepsin B functions as an innate immune factor against CD4-independent HIV-1 infection.

### Endosomal acidification is required for CD4-independent HIV-1 infection

The above results indicate that cathepsin B attenuates the CD4-independent mNDK vector infection in cells with high cathepsin B activity, but does not the CD4-dependent infection. Because cathepsin B is activated by low pH in acidic endosomes, the CD4-independent mNDK vector might enter host cells through acidic endosomes. Because the CD4-dependent HIV-1 does not enter host cells through acidic endosomes [25], cathepsin B might have no effect on the CD4-dependent infection. To examine these possibilities, the effects of endosome acidification inhibitors, bafilomycin A-1 (BFLA-1) and chloroquine, on the CD4-independent and –dependent HIV-1 vector infections were analyzed.

**Figure 9:**
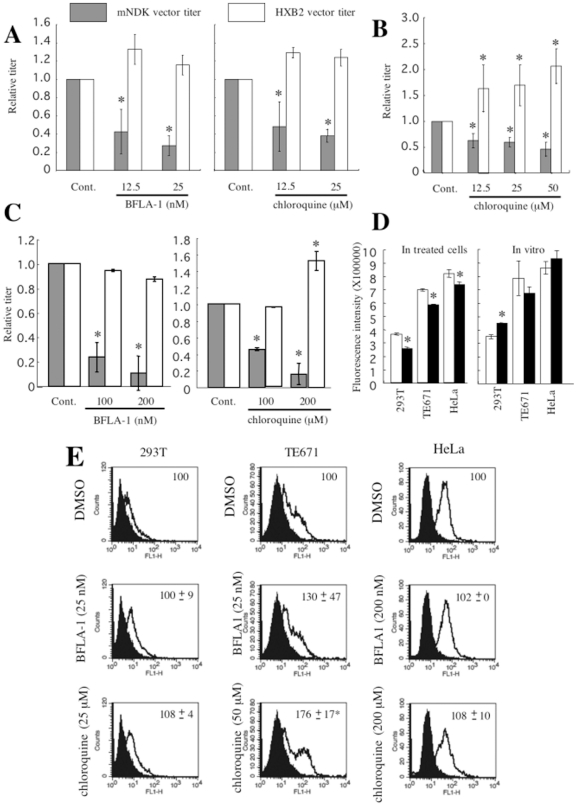
Effects of endosome acidification inhibitors, bafilomycin A-1 (BFLA-1) and chloroquine, on the mNDK or HXB2 Env-bearing HIV-1 vector. Transduction titers of the mNDK and HXB2 HIV-1 vectors were measured on 293T (panel A), TE671 (panel B), and HeLa (panel C) cells treated with the inhibitors. Relative values to the titers on cells treated with an equal volume of DMSO are indicated. Asterisks indicate statistically significant differences compared to the relative value on the DMSO-treated cells. Cathepsin B activities of cell lysates from the DMSO (open bar)- or chloroquine (closed bar)-treated cells were measured (panel D left panel). 293T, TE671, and HeLa cells were treated with chloroquine at 25, 50, and 200 µM, respectively. DMSO or chloroquine was added to cell lysates from untreated cells, and their cathepsin B activities were measured (panel D right panel). Fluorescence intensities were indicated. The cell surface expression of CXCR4 was analyzed on the treated cells by a fluorescence flow cytometer (panel E). Relative values to means of fluorescence intensity in the DMSO-treated cells are indicated. This experiment was repeated 3 times, and the results are shown as averages +− SD.

Target cells were pretreated with the inhibitors for 5 h before the HIV-1 vector inoculation. Treatment of 293T (Fig. 9A), TE671 (Fig. 9B), and HeLa (Fig. 9C) cells with these inhibitors attenuated the CD4-independent mNDK vector infection in a dose-dependent manner. In contrast, the CD4-dependent HXB2 (Fig. 9) and mNDK vector (data not shown) infections were not inhibited by these inhibitors in CD4-expressing cells, and were actually increased in TE671 and HeLa cells, as previously reported [25]. Higher concentrations of the inhibitors were required to inhibit the CD4-independent HIV-1 vector infection in HeLa cells than in 293T cells, correlating with the levels of cathepsin B activity in these cells. The chloroquine treatment of cells reduced cathepsin B activity of cell lysates prepared from the treated cells (Fig. 9D left panel). However, when chloroquine was added to the cell lysates, their cathesin B activities were not reduced (Fig. 9D right panel). Because cathepsin proteases are activated by endosome acidification, the endosome acidification inhibitor treatment of cells suppresses cathepsin B activation, but not cathepsin activity. Treatments with these inhibitors did not reduce the cell surface expression of CXCR4 (Fig. 9E), indicating that abrogation of the CD4-independent infection by the endosome acidification inhibitors is not due to the suppression of CXCR4 expression. These results indicate that the CD4-independent, but not the CD4-dependent, HIV-1 infection requires endosome acidification and occurs through acidic late endosomes.

### Endocytosis is required for CD4-independent HIV-1 infection

**Figure 10:**
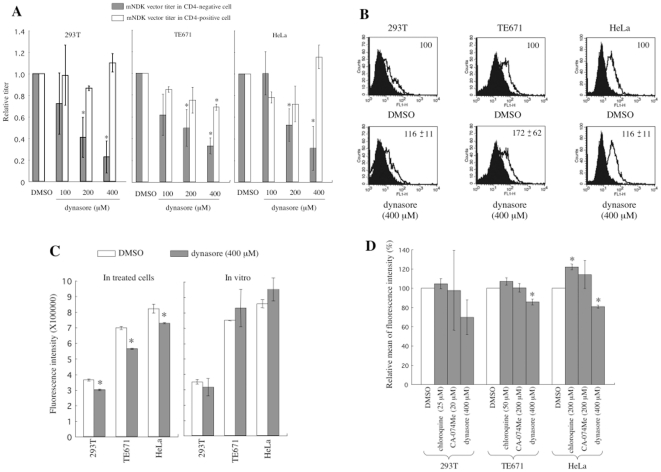
Effects of an endocytosis inhibitor, dynasore, on the mNDK Env-bearing HIV-1 vector infection. (A) Transduction titers of the mNDK HIV-1 vectors were measured on dynasore- or DMSO-treated 293T (left panel), TE671 (middle panel), and HeLa (right panel) cells. Relative values to the titers on cells treated with an equal volume of DMSO are indicated. (B) The cell surface expression of CXCR4 was analyzed on the treated cells by a fluorescence flow cytometer. Relative values to means of fluorescence intensity in the DMSO-treated cells are also indicated. (C) Cathepsin B activities of cell lysates from DMSO- or dynasore-treated cells were measured (left panel). DMSO or dynasore was added to cell lysates from untreated cells, and their cathepsin B activities were measured (right panel). Fluorescence intensities were indicated. (D) Effects of chloroquine, CA-074Me, and dynasore on endocytosis of Cy3-conjugated transferin were analyzed. Relative values to means of fluorescence intensity in DMSO-treated cells were indicated. These experiment were repeated 3 times, and the results are shown as averages +− SD.

The above results suggest that the CD4-independent HIV-1 particles are internalized by endocytosis for the infection. To assess this hypothesis, the effects of endocytosis inhibitors, dynasore or an Eps15 dominant negative mutant (Eps15-DN) [37], [38], on the CD4-independent and -dependent HIV-1 vector infections were analyzed. Target cells were pretreated with dynasore for 5 h. Dynasore inhibits dynamine GTPase and clathrin-dependent endocytosis [39]. Dynasore treatment more efficiently attenuated the CD4-independent mNDK vector infection than the CD4-dependent mNDK (Fig. 10A) or HXB2 (data not shown) vector infection in all examined cells (P<0.05). The dynasore treatment did not affect the cell surface expression of CXCR4 (Fig. 10B), demonstrating that dynasore treatment attenuated the CD4-independent HIV-1 infection through a mechanism other than the suppression of CXCR4 expression. The dynasore treatment of target cells moderately attenuated cathepsin B activity (Fig. 10C left panel). However, when dynasore was added to cell lysates, the cathepsin B activity was not affected (Fig. 10C right panel). Inhibition of endocytosis by dynasore might suppress cathepsin B activity by reducing number of endosomes in the treated cells, because active cathepsin B is present in late endosomes. The dynasore treatment inhibited endocytosis of Cy3-conjugated transferin as expected (Fig. 10D). However, the chloroquine or CA-074Me treatment did not. This result suggests that the chloroquine or CA-074Me treatment inhibits the CD4-independent HIV-1 vector infection by a mechanism other than endocytosis suppression.

**Figure 11:**
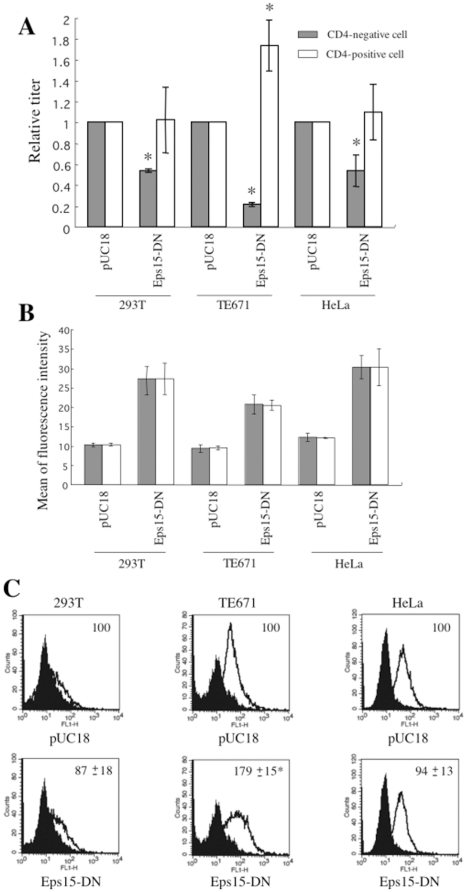
Dominant negative mutant of Eps15 inhibits CD4-independent HIV-1 vector infection, but not CD4-dependent infection. (A) Transduction titers of the mNDK vector were measured on 293T, TE671, and HeLa cells transfected with an expression plasmid of GFP-tagged Eps15 dominant negative mutant (Eps15-DN). Relative values to the titers on the pUC18-transfected cells were indicated. (B) The Eps15-DN expression in the transfected cells was analyzed by measuring fluorescence intensities of the transfected cells, because the Eps15-DN protein was tagged with GFP. Means of the fluorescence intensities of the transfected cells were indicated. Relative values to means of fluorescence intensity in the DMSO-treated cells are also indicated. (C) The cell surface expression of CXCR4 was analyzed on the treated cells by a fluorescence flow cytometer. Relative values to means of fluorescence intensity in the DMSO-treated cells are also indicated. These experiments were repeated 3 times, and the results are shown as averages +− SD.

Eps15 is involved in endosytosis, and its dominant negative mutant (Eps15-DN) suppresses endocytosis [37], [38]. Because the Eps15-DN is tagged with GFP, expression of the Eps15-DN in the transfected cells was analyzed by a flow cytometry. The Eps15-DN suppressed the CD4-independent mNDK infection, but not the CD4-dependent infection (Fig. 11A) under conditions that the levels of Eps15-DN expression in the CD4-negative and –positive cells were similar (Fig. 11B). In TE671 cells, the Eps15-DN rather enhanced the CD4-dependent HIV-1 vector infection provably due to elevated expression of CXCR4 (Fig. 11C). CXCR4 expression in 293T and HeLa cells was not affected by the Eps15-DN. These results indicate that endocytosis is more profoundly involved in the CD4-independent HIV-1 infection than the CD4-dependent infection.

### CD4-independent ROD/B vector infection also involves participation of endocytosis, endosomal acidification, and cathepsin B activities

**Figure 12:**
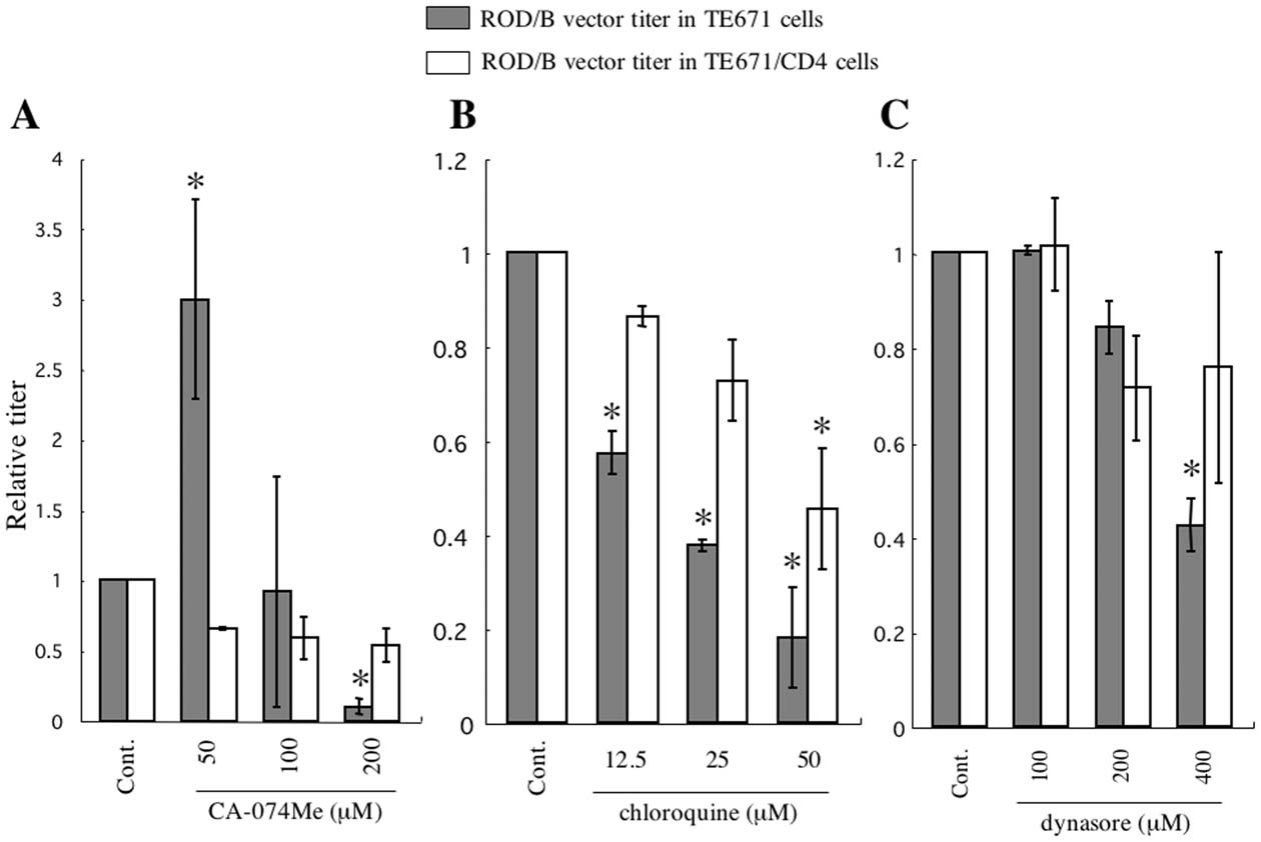
Effects of inhibitors of cathepsin B, endosome acidification, and endocytosis on CD4-independent ROB/B Env-pseudotyped HIV-1 vector infection. Transduction titers of the ROD/B vector were measured on TE671 and TE671/CD4 cells treated with CA-074Me (panel A), chloroquine (panel B), or dynasore (panel C). Relative values to the titers on the DMSO-treated cells (Cont.) are indicated. This experiment was repeated 3 times, and the results are shown as the means +− SD. Asterisks indicate statistically significant differences compared to the control cells.

The above results indicate that endosome acidification, endocytosis, and cathepsin B activity are involved in the CD4-independent mNDK Env-mediated infection. To assess whether these cellular events are also involved in another CD4-independent infection, HIV-1 vector containing Env protein of the CD4-independent ROD/B HIV-2 strain [40] was constructed. As seen in the mNDK infection, the ROD/B infection was enhanced by CA-074Me treatment at 50 µM in CD4-negative TE671 cells (Fig. 12A). The endosome acdification inhibitor (chloroquine) and endocytosis inhibitor (dynasore) suppressed the CD4-independent ROD/B infection, but not the CD4-dependent infection. These results indicate that endosome acidification, endocytosis, and cathepsin B activity are involved not only in the CD4-independent mNDK infection, but also in the CD4-independent ROD/B infection.

## Discussion

**Figure 13:**
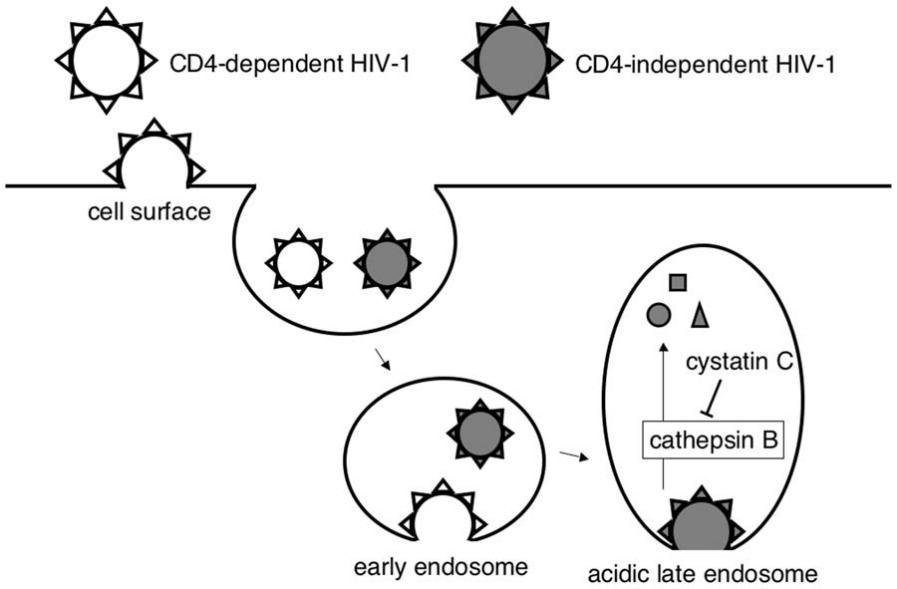
Infection route of the CD4-dependent (open) or CD4-independent (grey) HIV-1.

Inhibition of cathepsin B activity by cystatin C expression or by the specific inhibitor for cathepsin B, CA-074Me, significantly enhanced the CD4-independent HIV-1 vector infection in HeLa and TE671 cells, both of which express cathepsin B at relatively higher levels. Cathepsin B activity was inversely correlated with cellular susceptibility to the CD4-independent HIV-1 vector infection. Overexpression of cathepsin B attenuated the CD4-independent vector infection in 293T cells. The TLR4 signal activation also attenuated the CD4-independent vector infection by enhancing cathepsin B expression. These results indicate that cathepsin B is one of the host innate immune factors resisting to the CD4-independent HIV-1 infection. The endocytosis inhibitors, dynasore and Eps15-DN, suppressed the CD4-independent HIV-1 vector infection, showing that the CD4-independent infection occurs through endosomes. The mechanism by which cathepsin B inhibits the CD4-independent infection is suggested as follows; higher cathepsin B protease activity in SAOS-2, HeLa, and TE671 cells causes degradation of the HIV-1 particles in acidic endosomes, attenuating the CD4-independent HIV-1 infection (Fig. 13). Inhibition of cathepsin B by the cystatin C expression or by the CA-074Me treatment increases the CD4-independent infection by suppressing the viral particle degradation.

Since cystatin C is not expressed in 293T cells, cystatin C may not be the factor necessary for the CD4-independent infection in 293T cells. However, because cathepsin B clearly had an inhibitory effect on the CD4-independent infection and because cathepsin B activity in 293T cells is extremely low, 293T cells could have another factor(s) suppressing cathepsin B activity that is not expressed in HeLa cells. Another cystatin member, cystatin B, has been reported to be associated with higher susceptibility of monocyte-derived macrophages to CD4-dependent HIV-1 infection when compared to placental macrophages [41]. Because cystatin B is localized predominantly in the cytoplasm, while cystatin C is generally located in late endosomes [42] or secreted, and because cystatin C did not affect the CD4-dependent HIV-1 infection in our study, the roles of cystatins B and C in HIV-1 infection should be different.

Endosome acidification inhibitors are also known to attenuate infections by many other retroviruses, indicating that these retrovirus infections occur through acidic endosomes [13], [14], [15], [16], [17], [18]. In this study, the CD4-independent HIV-1 infection was attenuated by BFLA-1, chloroquine, dynasore, and Esp15-DN. This strongly indicates that the CD4-independent HIV-1 infection occurs through acidic endosomes, as in the case of other retroviruses (Fig. 13). In contrast, the inhibitors less efficiently attenuated the CD4-dependent HIV-1 vector infection than the CD4-independent infection, as they did in a previous report [25], suggesting that the CD4-dependent infection does not occur through acidic endosomes. However, Miyauchi et al. recently reported that CD4-dependent HIV-1 enters host cells via endosomes [26], [27]. The CD4-dependent HIV-1 particles might be internalized by dynamin-independent endocytosis. Even if the CD4-dependent HIV-1 particles are internalized into endosomes for productive infection, the CD4-dependent HIV-1 entry might occur before HIV-1 particle-bearing endosomes become acidic in late endosomes (Fig. 13). Further studies are needed to clarify this issue.

The endosome acidification inhibitors are thought to enhance the CD4-independent HIV-1 vector infection by suppressing cathepsin B activity, because cathepsin B is activated by low pH in acidic endosomes. Indeed, the endosome acidification inhibitors reduced cathepsin B activity. The inhibition of cathepsin B activity by the CA-074Me treatment enhanced the CD4-independent HIV-1 vector infection in HeLa and TE671 cells. Because the endosome acidification inhibitors much less efficiently attenuated cathepsin B activity than CA-074Me, the endosome acidification inhibitors could not enhance the CD4-independent infection. This result suggests that the endosome acidification itself is directly involved in the CD4-independent HIV-1 entry, as in the case of influenza virus and VSV [43].

TLR4 signal activation by the CD4-TLR4 attenuated the CD4-independent HIV-1 vector infection by enhancing cathepsin B expression, suggesting that cathepsin B functions as an innate immune factor resisting the CD4-independent HIV-1. Endosomal cathepsin proteases degrade microbes invading into host cells, and generate peptide presented on MHC class II in order to protect the host against the microbes [44]. In this context, it is interesting that several microbes encode cathepsin protease inhibitors homologous to the cystatin family [45], [46], [47]. Such microbial cystatins inhibit the host innate and adaptive immunity mediated by cathepsin proteases to allow efficient microbe proliferation in infected animals. The endocytic infection route of retroviruses might be changed to a cell surface infection route to escape from the host immunity mediated by endosomal cathepsin proteases, instead of having a cathepsin inhibitor.

This study indicates that cathepsin B inhibits CD4-independent HIV-1 infection. The CD4-independent HIV-1 infection occurs through acidic endosomes. The HIV-1 particles could be degraded predominantly by cathepsin B after the viral particles are internalized into endosomes, reducing the CD4-independent infection. Additionally, in this study, it was speculated that the CD4-dependent HIV-1 entry into host cells does not occur through acidic endosomes. Even when the CD4-dependent HIV-1 particles are internalized to endosomes, the viral entry might occur before the HIV-1 virion-containing endosomes have become acidic. Comparative studies between CD4-dependent and –independent HIV-1 infections will pave the way to clarification of the novel mechanisms that have not been disclosed using only CD4-dependent HIV-1, as we have already reported [28], [48].

## Materials and Methods

### Expression plasmids

The CD4-independent HIV Env (mNDK and ROD/B) expression plasmids were kindly provided by Dr. U. Hazan [4] and Dr. P. R. Clapham [49]. The CD4-dependent HIV-1 Env (HXB2 strain) expression plasmid was kindly provided by Dr. Y. Yokomaku. The VSV-G expression plasmid (pHEF-VSVG) was obtained from Dr. L. Chang [50] through the AIDS Research and Reference Reagent Program, Division of AIDS, NIAID, NIH, USA. An expression plasmid encoding the CD4-TLR4 fusion protein that is a constitutively active form of the TLR4 was constructed in our research group as previously reported [35]. An expression plasmid of the Eps15 dominant negative mutant [37], [38] was provided by Dr. A. Benmerah through Dr. M. J. Tremblay [51].

### Cells

Human HeLa [52], TE671 [53], 293T [54], SAOS-2 [55], and C33A [56] cells were cultured in Dulbecco's modified Eagle's medium (Wako, Osaka, Japan) at 37°C in 5% CO_2_. The culture media were supplemented with 8% fetal bovine serum (Biosource, Rockville, MD). HeLa, TE671, and 293T cells stably expressing CD4 (HeLa/CD4, TE671/CD4, 293T/CD4) were constructed as follows. These cells were transfected with an expression plasmid encoding CD4- and hygromycin-resistance genes (Invitrogen, Carlsbad, CA), and selected by hygromycin (Invitrogen). Hygromycin-resistant cell clones expressing CD4 were selected by FACS analysis using a FITC-conjugated anti-CD4 antibody (Sigma, St. Louis, MO). To construct cystatin C-expressing cells, HeLa, TE671, 293T, and their CD4-expressing cells were inoculated with an MLV vector encoding cystatin C (Origene, Rockville, MD) as previously reported [57]. Because the MLV vector additionally encodes a puromycin-resistance gene, the inoculated cells were selected by puromycin (Sigma). Puromycin-resistant cell pools were used in this study. To construct cathepsin B-overexpressing cells, 293T and 293T/CD4 cells were inoculated with a cathepsin B-encoding MLV vector, and selected by puromycin. The puromycin-resistant cell pools were used in this study. To construct hybridoma cells between HeLa and 293T cells, puromycin-resistant HeLa and geneticin-resistant 293T cells were fused by polyethylene glycol 1500 (Roche, Indianapolis, IN), and were selected simultaneously by puromycin and geneticin (Invitrogen). The double antibiotics-resistant cell pools were used in this study.

### Transduction assay

To obtain HIV-1 vector particles containing the Env protein, monkey COS7 cells were transfected with a packaging construct of HIV-1 (R8.91) [58], LacZ-containing HIV-1 vector (pTY-EfnLacZ) [59], and the appropriate Env expression plasmids (2 µg each) using the Fugene transfection reagent (Roche). The transfected cells were washed with medium 24 h after transfection, and continued to be cultured in fresh medium for 24 h. Target cells were pre-treated with chloroquine (Sigma), bafilomycin A-1 (BFLA1) (Sigma), CA-074Me (Sigma), or dynasore (Calbiochem, La Jolla, CA) for 5 h. Because these compounds were dissolved in DMSO (Sigma), cells were treated with an equal volume of DMSO as a control. The pre-treated cells were inoculated with the culture supernatants of the vector-producing cells. The inoculated cells were stained with 5-bromo-4-chloro-3-indolyl-β-D-galactopyranoside (X-Gal) (Wako) 2 days after inoculation. Blue cells were counted to estimate the transduction titer. The undiluted HIV-1 vector containing the mNDK Env protein induced (1.0+−0.4)×10^5^, (5.5+−3.1)×10^4^, (9.2+−4.4)×10^4^ blue cells in DMSO-treated 293T/CD4, TE671/CD4, and HeLa/CD4 cells, respectively. The undiluted mNDK HIV-1 vector induced (6.0+−1.6)×10^3^, (2.1+−0.5)×10^3^, and (2.1+−0.3)×10^2^ blue cells in DMSO-treated 293T, TE671, and HeLa cells, respectively. Therefore, when the HIV-1 vector was inoculated into CD4-positive cells, the vector solution was diluted 20-fold to normalize the transduction titer. The undiluted HIV-1 vector containing the ROD/B Env protein usually induced (5.3+−3.1)×10^4^ blue cells in CD4-expressing TE671 cells, and (1.9+−1.3)×10^4^ blue cells in original TE671 cells. Therefore, when the ROD/B vector was inoculated into CD4-positive cells, the vector was diluted 3-fold. DMSO-treatment did not affect these HIV-1 vector infections.

### Screening of the cDNA expression library

A cDNA expression library prepared from human lymph nodes was commercially obtained from TaKaRa (Shiga, Japan). HeLa cells were transfected with the cDNA expression library (3 µg) by using Fugene transfection reagent (50 µl), and were selected by geneticin (Invitrogen). The geneticin-resistant cell pools were inoculated with the blasticidin resistance gene-encoding mNDK Env-bearing HIV-1 vector. The HIV-1 vector was constructed by transfection of COS7 cells with the R8.91 and mNDK Env expression plasmids together with a blasticidin resistance gene-encoding HIV-1 vector genome expression plasmid (Invitrogen). The inoculated cells were selected by blasticidin (Invitrogen). Blasticidin-resistant cell clones were analyzed in this study. To amplify the cDNA sequence present in the blasticidin-resistant HeLa cells, PCR was performed using genomic DNA samples prepared from the cells. The nucleotide sequences of the PCR primers (Genenet, Fukuoka, Japan) were 5′-TAC GAC TCA CTA TAG GGA ATT CGA-3′ and 5′-TGT ATC TTA TCA TGT CTG GAT CCC-3′. Nucleotide sequence of the PCR product was determined by the Big Dye Terminator Cycle Sequencing Kit (Applied Biosystems, Warrington, UK). Accession number of the nucleotide sequence of the PCR product is AB587083.

### FACS

To analyze the cell surface expression of CXCR4, suspended cells were treated with a rat anti-CXCR4 antibody (A80) [60] or control serum for 1 h at 4°C. The cells were washed with PBS 3 times, and then treated with an FITC-conjugated anti-rat IgG antibody (Sigma). The stained cells were applied to a flow cytometer (BD Biosciences, San Jose, CA).

### Cathepsin B activity assay

To measure cathepsin B activity in living cells, cells were stained with the cathepsin B detection reagent (Cell Technology, Minneapolis, MN). This reagent utilizes fluorophore cresyl violet that is bi-substituted by an amide linkage to a peptide that contains a cathepsin B target cleavage sequence. In this form, the cresyl violet leaving group is non-fluorescent. Following cleavage at the amide linkage site by cathepsin B in living cells, the mono and non-substituted cresyl violet fluorophores generates red fluorescence. The stained cells were subjected to a flow cytometer (BD Biosciences) to measure their fluorescence intensity.

To measure the cathepsin B activity of cell lysates, a cathepsin B activity assay kit (BioVision, Mountain View, CA) was used. The cathepsin B activity assay kit is a fluorescence-based assay that utilizes the preferred cathepsin B substrate amino acid sequence labeled with amino-4-trifluoromethyl coumarin (AFC). Cell lysates that contain cathepsin B will cleave the synthetic AFC-labeled substrate to release free AFC, and generate fluorescence. Cell lysates were incubated with the cathepsin B substrate of the assay kit for 1 h at 37°C, and fluorescence intensity at 505 nm was measured by a fluorescence microplate reader (PerkinElmer, Turku, Finland).

### Western immunoblotting

Cell lysates were subjected to sodium dodecyl sulfate polyacrylamide gel electrophoresis (BioRad, Hercules, CA), and were transferred onto a PVDF membrane (Millipore, Billerica, MA). The membrane was treated with an anti-cystatin C or anti-actin antibody (Santa Cruz Biotechnology, Santa Cruz, CA), and then with a horseradish peroxidase-conjugated protein G (BioRad). Protein G-bound polypeptides were visualized by ECL Western blotting detection reagents (GE Healthcare, Buckinghamshire, UK).

### Semi-quantitative RT-PCR

Total RNA was isolated from cells by using TRIsol reagent (Invitrogen). First strand cDNA was synthesized from the total RNA with random hexamers by means of a reverse transcriptase (TaKaRa). Semi-quantitative PCR was performed using the first strand cDNA as a template to detect cystatin C, cathepsin B, and GAPDH mRNAs. The nucleotide sequences of the PCR primers (Genenet) for the cystatin C mRNA were 5′-TCC TAG CCG ACC ATG GCC-3′ and 5′-CGG TAC AGA CCC CTA GGC-3′, those for cathepsin B mRNA were 5′-GTG TAC CAA CAC GTC ACC GGA-3′ and 5′-CAG GCC CAC GGC AGA TTA-3′ [19], and those for the GAPDH mRNA were 5′-AGG TCG GAG TCA ACG GAT TTG GT-3′ and 5′-GTG GGC CAT GAG GTC CAC CAC-3′.

### Endocytosis assay

Target cells were pretreated with inhibitors for 4 h, and then starved in presence of inhibitors for 1 h. Cy3-conjugated transferin (4 µg/ml) (Jackson Laboratories) was added, and incubated for 10 min. Surface-bound transferin was removed by trypsin treatment of the cells for 15 min. Fluorescence intensities of the cells were measured by a flow cytometer (BD Biosciences).

### Statistical analysis

Differences between groups in relative values were determined by Student's *t*-test. Correlation between relative values of cathepsin B activities and susceptibilities to the CD4-independent mNDK vector infection was analyzed by Spearman's rank correlation. The level of statistical significance was set at p<0.05 for all tests.
